# Autistic preschoolers’ engagement and language use in gross motor versus symbolic play settings

**DOI:** 10.1177/23969415221115045

**Published:** 2022-07-27

**Authors:** Amanda V Binns, Devin M Casenhiser, Stuart G Shanker, Janis Oram Cardy

**Affiliations:** School of Communication Sciences and Disorders, 6221 Western University, London, Ontario, Canada; Bloorview Research Institute, Holland Bloorview Kids Rehabilitation Hospital, Toronto, Ontario, Canada; Audiology and Speech Pathology, The University of Tennessee Health Science Centre, Knoxville, Tennessee, USA; Department of Philosophy, 7991 York University, Toronto, Ontario, Canada; Communication Sciences and Disorders, 6221 Western University, London, Ontario, Canada

**Keywords:** Autism spectrum disorders, intervention/therapy, communication and language, parent-child interaction therapy, pre-school children

## Abstract

**Background and aims:** Although adjustment of the environment is recommended as a support strategy in evidence-based interventions for children with autism, the impact of doing so (and the how and why) is not well understood. One essential environmental factor to consider when providing supports for preschool-aged autistic children is the play setting, specifically, the materials available in the child's play context. The aim of this study was to compare engagement states and number of utterances produced by preschool-aged autistic children within symbolic vs. gross motor play settings. Examining the relationship between gross motor play settings and children's social engagement and spoken language use is particularly important to explore for autistic children given differences in their sensory processing, motor skill development, and choice of and interaction with toys relative to neurotypical peers. **Methods:** Seventy autistic children aged 25-57 months were videotaped during natural play interactions with a parent. Children's social engagement and number of spoken utterances were examined in five minutes each of play with symbolic toys and play with gross motor toys. Continuous time-tagged video coding of the child-caregiver engagement states was conducted, and the child's frequency of spoken language was identified using language sample analysis. The specific variables examined were; (a) engagement with caregiver, (b) engagement with objects only, (c) unengaged (no evident engagement with objects or people), and (d) total number of spoken utterances. The relationship between play setting (symbolic vs gross motor) and child language and engagement state variables was examined with linear mixed effects modelling. **Results:** Significant main effects were revealed for the interaction between play setting and autistic children's engagement. Young autistic children were more likely to engage with caregivers in play environments with gross motor toys (moderate effect) and also were more likely to have periods of unengaged time (not overtly directing their attention to objects or people; small effect) in this setting. Further, when in a setting with symbolic toys, autistic children were more likely to spend their time focusing attention solely on objects (large effect). No interaction was found between play setting and total number of utterances spoken by autistic children. **Conclusions and implications:** This study confirmed the importance of continued research focused on understanding the relationship between children's play settings and their social engagement and language use. Although preliminary, findings support the idea that there is an interaction between preschool-aged autistic children's social engagement and their play settings. Further, our results suggest that there can be value in clinicians differentiating children's play settings (i.e., gross motor vs symbolic) when assessing and supporting social engagement capacities of young autistic children.

The amount of time children spend engaged in social interactions is positively associated with their social communication and language development (e.g., Kasari et al., 2008; Kasari et al., 2012; Patterson et al., 2014; Tomasello & Farrar, 1986). This is because children’s capacity to socially engage facilitates their ability to link behaviours, experiences, or words with meaning, and develop social interaction competencies (Adamson, 1996; Bottema-Beutel et al., 2021; Bruner, 1983; Mundy & Jarrold, 2010; Nelson, 2007). Social engagement patterns between young autistic children and their caregivers consistently differ from their non-autistic and typically developing peers (e.g., Dawson et al., 2004; McArthur & Adamson, 1996). For example, studies have shown that preschool-aged autistic children are more likely than their age-matched peers to spend longer durations attending to objects, and less time engaged with partners in their play environments (Adamson & Chance, 1998; Dawson et al., 2004; Swettenham et al., 1998). Autistic children also tend to engage in and initiate joint attention less frequently than age-matched peers (e.g., Dawson et al., 2004; Werner & Dawson, 2005). Given the differences in social engagement patterns and the links between social engagement and spoken language development (e.g., Kasari et al., 2008; Patterson et al., 2014), it is not surprising that studies have also revealed that preschool-aged autistic children produce fewer spoken utterances than age-matched typically developing peers (e.g., Tek et al., 2014). With children’s early language functioning strongly correlating with long term social-wellbeing outcomes (Szatmari et al., 2009; Tager-Flusberg et al., 2005; Tidmarsh & Volkmar, 2003; Venter et al., 1992), improving our understanding of factors that may support opportunities for autistic children to build foundational social engagement capacities and language is critical. Therefore, the purpose of this study is to explore the relationship between play settings and autistic children’s engagement states and use of language.

## Social engagement

Social engagement is often a treatment target within caregiver mediated programs designed to support autistic preschool-aged children and their caregivers (e.g., Brian et al., 2017; Greenspan & Wieder, 2006; Kasari et al., 2008; Solomon et al., 2014; Sussman et al., 2016). Behavioral signs of social engagement may include: social referencing, affective exchanges, joint attention, and reciprocal communicative exchanges; however, although it is parsimonious to describe and measure engagement as discrete child behaviours, it is important to consider the fundamentally dyadic nature of engagement. Particularly during interactions with young children, caregivers provide scaffolding to establish and maintain their children’s attention to people or objects, and to support children to co-ordinate attention and reciprocally engage (Bakeman & Adamson, 1984; Bruner, 1983). To capture the dyadic and extended nature of social engagement in the context of play with toys, Bakeman and Adamson (1984) suggested identification of *engagement states*. Types of engagement states include interactions solely with caregivers (child + caregiver) or objects (child + object), and more complicated joint engagement states (child + caregiver + shared referent). Children’s capacities to reciprocally engage solely with people (child + caregiver) through use of expressive affect and gestural communication, and engage solely with objects (child + object) are thought to be foundational for developing more complicated forms of coordinated joint engagement (i.e., child + caregiver + object; Adamson & Chance, 1998; Brazelton, Koslowski & Main, 1974; Bruner, 1983; Kaye & Fogel, 1980) and, subsequently, language (e.g. Crandall et al., 2019).

## Social engagement, language, and play settings

According to transactional and systems theories of development (Sameroff & Fiese, 2000; Thelen & Smith, 1994), children’s social interactions and use of language manifest differently depending on the social context, which can include the environment, materials available, and familiarity of play partners (e.g. Abbeduto et al., 1995; Frost et al., 2019; Kover et al., 2014; Miles et al., 2006; O'Brien & Bi, 1995). This has implications for professionals assessing and supporting parent-child engagement and language in autistic children. Indeed, adjustment of the environment is a key support strategy used in both Developmental Social Pragmatic and Naturalistic Developmental Behavioural Interventions (Binns & Oram Cardy, 2019). However, specific information regarding the impact of making environmental adjustments (and the *how*, and *why*) is not well understood.

When providing supports for preschool-aged children, one essential environmental factor to consider is the play setting, specifically, the materials available in the children’s play context.

O'Brien and Bi (1995) examined teacher and child language across play settings. More specifically, they examined the frequency and nature of non-autistic preschool children’s spoken language use (*M* = 25 months) across three play settings (doll house, block/truck, and gross motor areas) within a real-world classroom. Their findings revealed that different types of play (i.e., symbolic vs. gross motor) yielded very different language output from young children. Children in their study spoke more often and used more complex language during symbolic play as compared to gross motor play. They also found that different toys within the same type of play (i.e., blocks vs. dolls and food) yielded different language from children, with children using more statements and fewer labels during symbolic play with open-ended toys such as blocks and toy cars, as compared to play with dolls and a play house. Additionally, they found that teachers’ language use patterns significantly differed across play contexts, and these were associated with differences in the rate and nature of child language. Few studies, however, have empirically examined the relationship between play settings and young autistic children’s social engagement and spoken language. Even fewer have looked specifically at the relationship between gross motor play settings and autistic children’s engagement and spoken language.

Such examination of the relationship between gross motor play settings and children’s social engagement and spoken language use may be particularly important to explore for autistic children given their sensory processing differences (Robertson & Baron-Cohen, 2017), differences in motor skill development (Flanagan et al., 2012), and reported differences in choice and interaction with toys (i.e., using gross motor toys more often than neurotypical peers; Dominguez et al., 2006). MacDonald and colleagues (2017) compared 2- to 7-year-old autistic and non-autistic children’s engagement, sustained attention, and connectedness with their caregiver across two parent-child play sessions: a traditional social play setting and a motor-based-behaviour setting. In the traditional play setting, children were presented with toys such as miniature characters, objects that could be used imaginatively (e.g., strings and blocks), cars, cause and effect toys, and building/construction toys. Materials available to the children in the motor-based-behaviour setting included miniature stairs, mats, wedge mats, slides, teeter-totter, balance beam, tunnel, tricycle, balls, and targets. Results revealed significantly lower engagement, sustained attention, and level of connectedness with their parent in the motor behaviour-based play setting for the autistic children as compared to their neurotypical peers. Within the social play setting, autistic children and their peers performed similarly, with the exception of engagement, which remained significantly lower for autistic children compared to their peers. This suggested that children with autism have less engagement with their parent or caregiver than their typically developing peers across both motor and social play settings, although fewer group differences were observed in the latter. Swettenham and colleagues (1998) and Adamson and colleagues (2016) also found that autistic children were more likely than their age matched peers to spend longer durations attending to objects, and less time attending to people in their play environments. Together, these studies provide insight into the differences of social engagement and language use patterns across different populations (autistic vs. non-autistic children); however, they did not explore the relationship between play contexts and engagement states or language across the same population (autistic children). Therefore, there remains a gap in the literature that could be used to inform clinicians about how they could tailor autistic children’s play contexts to support social engagement and spoken language use.

## The current study

Extending the current body of literature, our study aimed to better understand the relationship between play settings and children’s engagement states and use of spoken language – specifically within the autistic population. We accomplished this by comparing preschool-aged autistic children’s engagement states and number of utterances produced when in symbolic versus gross motor play settings. Strong correlations between autistic children’s engagement states or spoken language use and their play contexts would justify further investigation in this currently understudied area. Furthermore, findings could provide new, preliminary insights into *how* autistic children engage and communicate across symbolic and gross motor play contexts that could be used to inform clinical decision making. Specific research questions included:

- Do autistic preschool-aged children differ in the proportion of time they spend engaged with objects, engaged with their caregivers, or unengaged across play settings with gross motor toys versus symbolic toys, when controlling for age?
- Do autistic preschool-aged children differ in the number of utterances they produce across play settings with gross motor versus symbolic toys, when controlling for age?

Informed by social cognitive theories of development (which suggest environments can impact children’s use of language), and what is known about the relationship between play settings and the frequency of spoken language and social engagement patterns of non-autistic children (O'Brien & Bi, 1995), we hypothesized there would be statistically significant differences in the proportion of time spent across engagement states, and the number of spoken utterances used by autistic children across gross motor versus symbolic play settings. However, because of the limited empirical evidence about child engagement states and spoken utterances specifically in gross motor versus symbolic play settings, we did not hypothesize a direction of these relationships. Yet, given the strong relationship between children’s social engagement (i.e. engagement with caregivers) and use of spoken language (e.g., Kasari et al., 2008; Patterson et al., 2014), we anticipated similar directional patterns for these two variables.

## Method

### Participants

Participants included 70 children (and parents) who were recruited through diagnosing physicians, public service agencies, and newspaper advertisements in the Greater Toronto Area. Of the 70 participants, 51 participated in a previously reported randomized control trial (Casenhiser et al., 2013; Casenhiser et al., 2015). Children met the following criteria prior to study entry: (a) clinical diagnosis of autism spectrum disorder, confirmed by the Autism Diagnostic Observation Schedule (ADOS) and Autism Diagnostic Interview, (b) chronological age between 2 years 0 months and 4 years 11 months, and (c) no secondary neurological or developmental diagnoses (e.g., seizure disorder, global developmental delay; Casenhiser et al., 2015). Parents who enrolled in Casenhiser and colleagues’ study committed to attend a 2-h session weekly for a period of 12 months, and spend an additional 10–13 h per week implementing therapy strategies at home. Demographic information is presented in Table 1.

**Table 1.** Demographic information of participants.

Demographic variables	Overall (n = 70)
Chronological Age Child (months) Mean Median Range	42.5 44.0 25.0–57.0
Sex, n (%) Female Male	5 (7%) 65 (93%)
Parent sex, n (%) Female Male	54 (77%) 16 (23%)
Family income*	51% (over 100 000) 22% (50 000-100 000) 27% (less than 50 000)
Mother’s education level**	16% (advanced degree) 52% (bachelors degree) 8% (associates degree) 22% (some university/college) 4% (high school)
Language most often spoken at home English Other	62 8

*Incomes are reported for 46 families and are in Canadian dollars. Six families elected not to provide information on their income, and family income was not available for 18 of the families. Statistics Canada reports the 2008 median gross income in Canada is approximately $76,000 (2010).

**Mother’s education level was reported for 52 families and was not available for 18 families.

To characterize our sample of participants, scores from the ADOS (APA, 2013), cognitive age equivalent scores (taken from Wechsler Preschool and Primary Scale of Intelligence, and Bayley Scales of Infant and Toddler Development) and language age equivalent scores (from the Preschool Language Scale and the Comprehensive Assessment of Spoken Language) are presented in Table 2.

**Table 2.** Participant characteristics.

	Mean	*SD*	Range
ADOS (raw score)	15.03	3.68	7-21
Cognitive age equivalent (month)	33.76	11.06	14–56
Language age equivalent (months)	21.59	12.33	2–60

ADOS: autism diagnostic observation schedule; *SD*: standard deviation.

### Overview of design and procedures

Institutional review board approval was obtained prior to enrollment of participants and all families provided informed consent to participate in this research. A repeated measures design was used for this study. To collect data on children’s engagement states and number of spoken utterances across two play settings, we used a set of pre-treatment, videotaped, caregiver-child interactions. Videos were collected in a research laboratory setting at York University in Toronto, Canada. The entire caregiver-child, free-play interaction was 25 min and consisted of 15 min of access to symbolic toys, 5 min of access to tactile toys, and 5 min of access to gross motor toys, presented in this same order for all participants. For the purpose of the present analysis, we elected to examine the first codable 5 min of the symbolic toy section and the 5-min gross motor toy section. We used only 5 min of the symbolic section so that the amount of time was the same across both play settings. Prior to being videotaped, caregivers were instructed to play with their child as they would at home. They were then presented with the different sets of toys. The symbolic toys included toy food, a shopping cart, a cash register, a toy house, toy cars, and puppets. Gross motor toys included a crash mat, small trampoline, exercise ball, and spinning desk chair.

### Coding and reliability

#### Engagement state variables

Continuous time-tagged video coding of the children’s engagement states was conducted using Datavyu software (Datavyu Team, 2014). This entails marking the start and stop of three mutually exclusive engagement state codes throughout a video, so a total duration for each state can be calculated for each participant in both a gross motor and a symbolic play setting video. Five minutes of codable videos were analyzed for each participant. Moments in which the child was crying or the child’s body was offscreen were considered *uncodable* and were not included within the 5 min samples analyzed. Coding procedures were informed by Adamson and colleagues’ (2000) engagement state coding system. Three engagement state variables were examined: engagement with caregiver, engagement with objects only, and unengaged (no evident engagement with objects or people). States had to last for at least 3 s to be coded. The variable *engagement with caregiver* involves the child attending to social stimuli, and is inclusive of Adamson’s and colleagues (2000) engaged with caregiver code and joint attention codes (i.e., supported joint attention, co-ordinated joint attention, and symbol infused joint attention states). We elected to collapse these engagement state codes because of the young age of our sample and because we were interested in evaluating children’s overall social engagement with parents. Both children’s social orienting and joint attention behaviours are highly correlated, suggesting that they measure a common construct (Dawson et al., 2004). Descriptions and examples of each of the engagement codes appear in Table 3.

**Table 3.** Descriptions and examples of engagement variables based on Adamson et al. (2000).

Engagement variables	Explanation	Examples
Engaged with Caregiver	Engagement with caregivers was defined as children’s time spent: watching/observing caregiver, engaged with a caregiver (with only minimal involvement of toys), engaged in social referencing (responding to, and initiating, using social referencing and/or verbal referencing).	Child watching parent jump on the trampoline while waiting for a turn People play, such as the child and caregiver making a game of the child jumping into the caregiver’s arms Caregiver demonstrates how to use a toy, child watches then spontaneously imitates actions to use toy The child bangs their hand onto the same toy that the caregiver is manipulating it, and then looks at the caregiver, bangs the toy, and then looks back at the caregiver, smiling
Engaged with Objects	The child is visually attending to an object, exploring or playing with it independently. The caregiver may attempt to engage the child, but the child ignores them. Segments in which the child is merely in contact with an object, as when they hold a small toy while scanning the room (not visually or auditorily attending to the toy) are not included.	Child focuses attention on spinning wheels on a chair Child visually explores the lines on the side of a doll house. Child focuses attention solely on toy figurine.
Unengaged	No apparent engagement with a specific person, object, or symbols. The child may be unoccupied, may be scanning the environment as though looking for something with which to be engaged, or may be flitting between foci without committing to any.	Child walking the perimeter of the room Child sitting independently and using self-talk without directing it to caregiver or shifting gaze toward caregiver.

Three graduate students in speech-language pathology were trained on the coding system by the first author over 3 months and were not aware of this study’s specific research questions or hypotheses at the time of coding. Double coding for 40% of the videos across both play settings was conducted, with the same coder coding both the symbolic and gross motor play setting videos for a participant. Cronbach’s alpha was used to calculate internal reliability (Casenhiser et al., 2015); above 0.70 is considered acceptable, however, greater than 0.80 is preferred (Cortina, 1993). Internal reliability for the set of engagement codes in the symbolic play setting was strong: Cronbach’s α = .850, and was similar for engagement codes in the gross motor setting: Cronbach’s α = .830. All disagreements were discussed until 100% agreement was reached.

#### Language variable

Videos were transcribed by C-units and examined using the Child Language Data Exchange System (CHILDES; MacWhinney, 2000). The kidEVAL program was used to calculate number of utterances. Children’s reciting of songs or poems and exact repetitions of their own previous utterances without a change in context were excluded when calculating total number of utterances produced. The mean proportion of utterances that were excluded for this reason in the gross motor play setting was 9.93% (*SD* = 21.27), and in the symbolic play setting was 12.66% (*SD* = 18.84). Transcription reliability between trained graduate students in speech-language pathology and trained undergraduate research assistants was computed for 25% of the participants and percent agreement for utterance boundaries across transcripts was 96%.

#### Analytic methods

To address our research questions, the relationship between play setting (symbolic vs. gross motor) and number of utterances and engagement variables was examined with linear mixed effects modeling using R (R Core Team, 2020) and the lme4 package (Bates et al., 2012). This method was selected because linear mixed effect models are relatively robust against violations of the assumptions of normality (Gellman & Hill, 2007), and they allow for the resolution of non-independencies in our data (Winter, 2013). Using liner mixed effect models, we are able to depict the relationships between play settings and the engagement and language variables while properly accounting for the within-subject factor. That is, by including *participant* as a random effect in the linear mixed effects model, the idiosyncratic variation due to individual differences across participants is characterized. The assumption is that each participant has a unique intercept for each variable. Given the heterogeneity across autistic children, it is particularly advantageous to control for this individual variation among participants.

With consideration given to individual differences across autistic children, pirate plots were used to visualize descriptive statistics for all dependent variables. This data visualization method is thought to be *data rich* and *data accountable*, meaning all data points are presented and there is more transparency and credibility in the analyses (Larson-Hall, 2017).

## Results

We ran separate models for each of our dependent variables. A Wald test was used to compare the fit on successive models. Statistical significance of the fixed effect *play setting* was obtained by testing the full model with the effect in question against the null models (without the effect in question) using the Akaike Information Criterion. This allowed for arbitrating the explanatory power of the models. Systematic visual inspection was used to examine homoscedasticity and normality of the residuals. As expected, there was a significant effect of age on children’s overall social engagement, engagement with objects, periods of engagement, and number of spoken utterances (see Table 4). However, the best model fit for all dependent variables in question included age + context entered as fixed effects. Adding an interaction between age and play setting did not improve model fit for: engagement with caregiver (*p* = 0.130), object engagement (*p* = 0.241), unengaged (*p* = 0.124), or number of spoken utterances (*p* = 0.937).

**Table 4.** Random and fixed effects parameters for all four mixed models.

Model	Fixed effects	Random effects	Estimate	SE	*t*	*p* value	Variance	SD
Engagement Models								
Time (seconds) engaged with caregiver	Intercept		215.829	9.12	20.05			*** ***
	Age (3yrs)		−32.40	17.73	−1.83	0.0719		*** ***
	Age (2yrs)		−74.26	68.96	−3.51	0.0008*		*** ***
	Setting (Gross-Sym)		−26.76	8.72	−3.06	0.0032*		*** ***
		Subject		*** ***	*** ***		2610	51.09
Time (seconds) engaged with objects only	Intercept		41.61	11.72	3.55			
	Age (3yrs)		11.02	13.58	0.81	0.420		
	Age (2yrs)		34.63	16.19	2.14	0.036*		
	Setting (Gross-Sym)		41.98	8.56	4.903	<.0001*		
		Subject		*** ***	*** ***		1016	31.87
Time (seconds) unengeged	Intercept		28.93	7.52	3.85			
	Age (3rs)		20.54	8.30	2.47	0.0158*		
	Age (2yrs)		39.06	9.89	3.95	0.0001*		
	Setting (Gross-Sym)		−17.49	6.94	−2.52	0.0139*		
		Subject		*** ***	*** ***		14.04	3.75
Language Models								
Total Utterances	Intercept		36.45	4.10	8.90			
	Age (3yrs)		−15.93	5.03	−3.16	0.0023*		
	Age (2yrs)		−30.02	6.02	−4.99	>0.000*		
	Setting (Gross-Sym)		−1.24	1.53	−0.81	0.4222		
		Subject					385.94	19.65

*Note.* Gross = gross motor toy setting, Sym = symbolic toy setting; Within R, items are coded alphabetically, therefore the gross motor setting was coded as 0, and the symbolic setting coded as 1; Similarly, ‘age’ reference group is 4yrs, so the coefficient corresponds to the effect of age on children’s overall engagement states/number of utterances compared to group of 4yr old children.

Age (as a factor: 2yrs, 3yrs, 4yrs) and play setting (gross motor or symbolic) were entered into the model as fixed effects, and all models were built with participants entered as a random effect (random intercept). Significant interaction effects were further explored using post-hoc pair-wise comparisons across participants and context, provided by the *emmeans* package (Lenth et al., 2018), with Holm- Bonferroni adjustment for family-wise error. Effect size (eta-squared) for each model was calculated using the *anova stats* function, provided by the *sjstats* package (Lüdecke, 2020).

**Figure 1.**
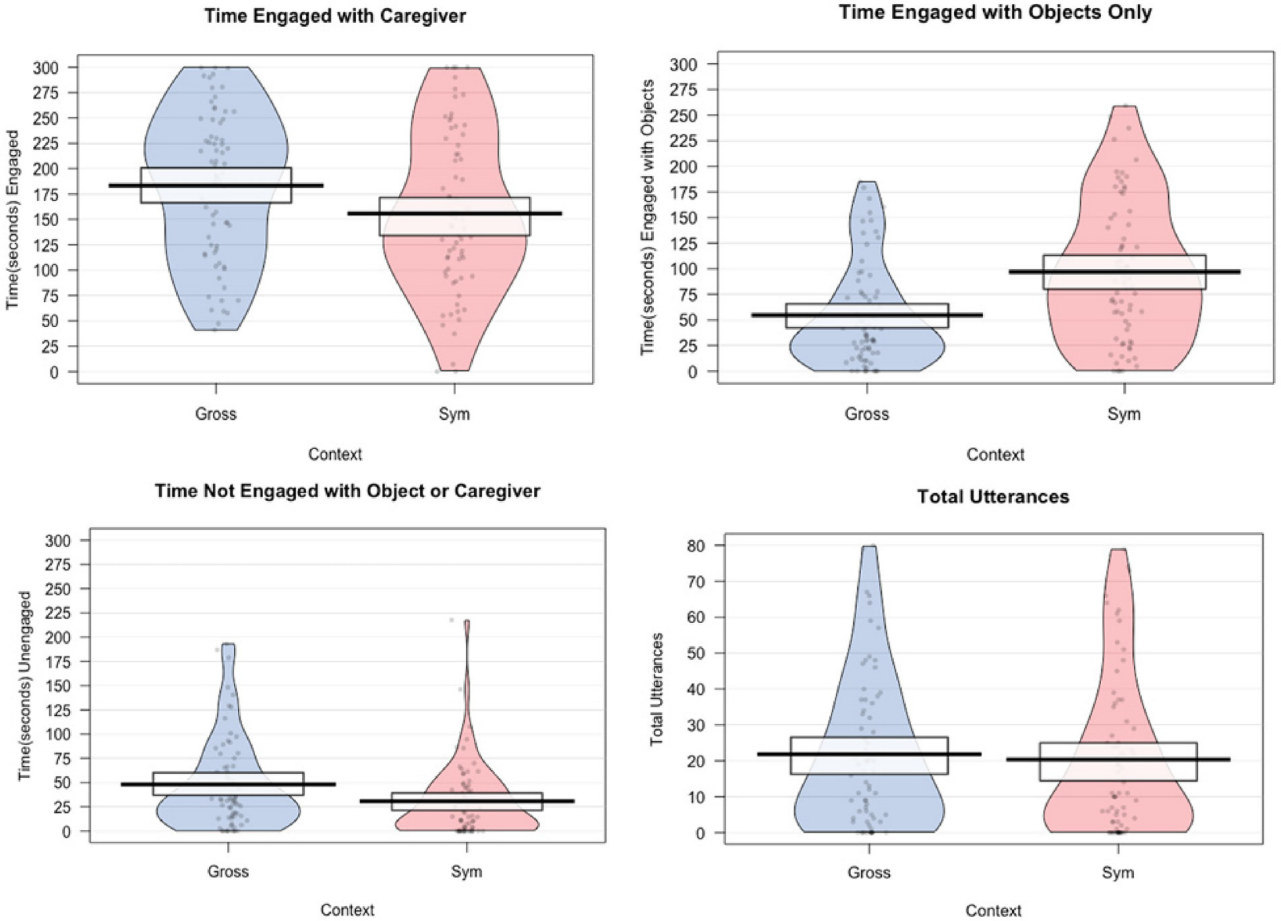
Pirateplots of descriptive data (group means, 95% confidence intervals) for engagement states and number of utterances across symbolic and gross motor play settings. *Note.* Gross = Gross Motor Setting; Sym = Symbolic Setting.

Figure 1 presents pirate plots of the descriptive statistics for all dependent variables. Each pirate plot presents visualization of raw data, distribution of means (via smoothed density curves), and 95% confidence intervals. The interaction of play setting and children’s engagement and language variables was examined using linear mixed effect models, with setting entered as a fixed effect and participant entered as a random effect. Separate models were created for each dependent variable. Table 4 presents random and fixed effects parameters for all four models. Systematic visual inspection of residual plots for each model did not reveal any obvious deviations from homoscedasticity or normality. Table 5 presents the estimated marginal means for each model.

**Table 5.** Estimated marginal means for each model.

Variable	Symbolic setting	Gross motor setting
Time (seconds) Engaged with Caregiver		
Estimated Marginal Mean (Standard Error)	154 (9.02)	180 (9.02)
Df	106	106
95% confidence level	136–171	162–198
Time (seconds) Engaged with Objects Only		
Estimated Marginal Mean (Standard Error)	98.8 (7.4)	56.8 (7.4)
Df	122	122
95% confidence level	84.2–113.5	42.2–71.5
Time (seconds) Unengaged		
Estimated Marginal Mean (Standard Error)	31.3 (5.05)	48.8 (5.05)
Df	135	135
95% confidence level	21.3–41.3	38.8–58.8
Total Number of Utterances		
Estimated Marginal Mean (Standard Error)	19.9 (2.37)	21.1 (2.37)
Df	83.7	83.7
95% confidence level	15.2-24.6	16.4-25.9

### The interactions between play settings and engagement states

Examination of the impact of play setting (gross motor vs. symbolic) on children’s engagement with their caregiver (while controlling for age) revealed significant main effect, F(1, 69) = 9.40, *p* = 0.003, with a moderate effect (η^2^ = 0.093). Within the symbolic toy setting, there was less time spent engaged with their caregiver by 26.76s ± 8.73 (SE), as compared to the gross motor context. In other words, when in a symbolic play setting for 5 min, children spent roughly 9% less time engaged with their caregiver than they did during the gross motor play setting. Play setting also had an impact on children’s engagement solely with objects F(1, 69) = 24.04, *p* = 0.001, with a large effect (η^2^ = 0.185). During the 5 min of play within the symbolic play setting, we saw children engage with objects for 41.98s ± 8.56 (SE) more than compared to their attention to objects in the 5-min gross motor setting. This is 14% more time spent engaged with objects only during the symbolic play setting. Finally, a significant main effect was also revealed for the relationship between play setting and children’s time spent unengaged (with objects or people) F(1, 69) = 6.42, *p* = 0.014, with a small effect (η^2^ = 0.045). On average, children spent less time unengaged by 17.50s ± 6.936 (SE) when in the symbolic setting as compared to the gross motor play setting.

### The impact of play setting on number of spoken utterances

No interaction between play setting and the total number of utterances spoken by children was found F(1, 68) = 0.652, p > 0.05. The mean number of children’s utterances in the gross motor play setting was 21.8, and the median was 17 (Range: 0–80). During the symbolic play context, the mean number of utterances was 20.6, and the median was 11 (Range: 0–79). Children produced an average of 4 utterances per minute across both play settings.

## Discussion

Our study set out to better understand the relationship between play settings and preschool aged children’s engagement states and use of spoken language – specifically within the autistic population. Our findings extend the existing literature that has more broadly explored the interactions between autistic children’s play environments and social communication patterns (e.g., Frost et al., 2019; Kover et al., 2014), or compared social communication patterns of autistic children to non-autistic peers (e.g., MacDonald et al., 2017), by comparing preschool aged autistic children’s proportion of time spent in engagement states and their frequency of spoken language across gross motor and symbolic play contexts. There were individual differences across the children in our sample. Nonetheless, in alignment with our general hypothesis, the proportion of time our sample of pre-school aged autistic children engaged with their caregivers, engaged with objects, and were unengaged significantly differed across gross-motor versus symbolic play contexts. Yet, we did not find significant differences between the autistic children’s number of spoken utterances across contexts. Nonetheless, the fact that our group of autistic children’s frequency of spoken language did not decrease within the gross motor setting relative to the symbolic toy setting (as is reported for non-autistic children; O'Brien & Bi, 1995) is of interest, and may be related to more time spent engaged with caregivers in the gross motor setting. Taking into account previous studies conducted with non-autistic peers (e.g. O'Brien & Bi, 1995), our findings suggest that autistic children’s communication patterns across gross motor and symbolic play settings may follow different directional patterns than non-autistic peers.

In the following sections we will explore the *how* and *why* of our findings by highlighting several possible explanations for our results and sharing potential clinical implications and future directions for research.

### Engagement state differences across play settings

Autistic children in our study spent less time focusing on objects only, more time engaged with caregivers, and slightly more time unengaged (with objects or people in their environment), during the gross motor play setting relative to the symbolic play setting. Findings may be related to a number of factors that have yet to be tested but are worthy of consideration. One possibility is that this was merely an artefact of the order in which the play settings were presented, that is, children may have engaged more with caregivers in the gross motor play setting because this context was always presented to the child after the symbolic setting. It is plausible that children were *warming up* during the symbolic play setting, and that they spent more time engaged with caregivers in the gross motor setting because they were becoming more comfortable with the environment over time. However, the data from our symbolic play setting is closely aligned with data on young autistic children’s engagement states within semi-natural play interactions from Adamson et al. (2012). This suggests that although it is possible that children may have interacted differently in the symbolic play setting as a function of the order in which the two contexts were filmed, the patterns of children’s engagement observed in our symbolic play setting appear to be representative of young autistic children’s engagement in symbolic contexts.

It could also be the case that the properties of the toys provided in each of the play environments, rather than the nature of the toys (symbolic vs. gross motor), contributed to our findings of systematic differences in children’s engagement across play contexts. For the toys used in this study, these properties include how they are used, their size, and the degree of visual detail within them. Generally, the symbolic and gross motor toys used in our study are designed to be used very differently. For young children, the gross motor toys may be more likely to require a partner’s assistance for use than the symbolic toys. For example, in the gross motor setting a child could jump on a crash mat and might require caregivers to hold their hands for stabilization, while in the symbolic setting they could explore a toy car and figurine independently. It could be the case that the *built in* need for caregiver’s assistance to use many of the gross motor toys in our study contributed to children’s attention being directed more toward caregivers in the gross motor context. We also speculate that the open-ended and (perhaps) less familiar nature of the gross motor toys contributed to children’s longer proportion of time spent unengaged in the gross motor play context as compared to the symbolic play context. For example, a crashmat, large yoga ball, and spinning chair could all be used in many different ways, and these types of toys may not be routinely found in children’s toys at home. Given that autistic children may experience difficulties in motor-planning (Flanagan et al., 2012), it may have taken them longer to generate and execute their idea for engaging with these toys, and thus contributing to more time unengaged in the gross motor play context. Qualitative analysis examining which of the toys the children spent most time playing with across the different play settings, who initiated the play ideas with the toys, and exploration of how play with different toys aligned with children’ engagement states would be interesting to explore in future studies.

Additionally, there was a distinct difference in the size of the toys provided in the symbolic versus the gross motor play setting. Toys in the gross motor play setting were much larger (i.e., personal trampoline, crash mat, large yoga ball, spinning chair) than the toys provided in the symbolic play setting (i.e., action figures, small toy cars, play food items). We speculate that the size and amount of visual detail on the toys in the environment may have implications for autistic children’s social engagement patterns. This viewpoint aligns with the idea that autistic children pay attention to people and objects in their environments differently than neuro-typical peers (e.g., Elsabbagh et al., 2011; Remington et al., 2009), and that these perceptual difference may lead autistic individuals to be more detail-focused and distracted by visual details of objects, which may also make it harder for them to *zoom out* their attentional focus and shift attention to social stimuli when playing with small. visually detailed symbolic toys (Robertson et al., 2013; Ronconi et al., 2013). When children are playing with larger toys, they are more likely to be positioned in a more upright posture. Thus, their visual field is likely to be expanded (zoomed out), potentially making it less effortful for them to shift their focus of attention toward their play partners. Moreover, the toys in the gross motor setting also tended to have less visual detail than the toys presented in the symbolic play setting. For example, a large yoga ball (gross motor toy) has less complex visual details than a cat figurine with whiskers, stripes, etc. (symbolic toy). Thus, when children were in the symbolic play setting, their attention could have been more *zoomed in* and focused on the objects in their environment (rather than their caregivers) because the toys tended to be more visually detailed/interesting. Systematic testing of the impact of the aforementioned toy properties on children’s engagement states should be explored in future work, to form a more detailed understanding of the impact of the properties of toys (i.e., size, amount of visual detail) on young autistic children’s social engagement.

The final factor to consider when interpreting the engagement results revealed in our study relates to the impact gross motor play activities can have on children’s arousal level. We know from listening to the lived experiences of autistic self-advocates and empirical research that autistic children have sensory-regulatory differences that can impact arousal (e.g., Baranek et al., 2007; Baranek et al., 2013; Cascio et al., 2016; Fletcher-Watson & Happé, 2019; Welch et al., 2020). In addition, there is evidence indicating a relationship between arousal and behaviours linked to social engagement, such as attention shifting and re-orienting (e.g. Marrocco & Davidson, 1998; Orekhova & Stroganova, 2014). Furthermore, gross motor play requires physical exertion and thus is likely to increase children’s arousal levels more so than symbolic play. Therefore, the toys provided to children during gross motor play could have been upregulating children’s arousal level, potentially making it easier (less effortful) for them to shift attention. In future work, adding a measure to examine children’s arousal during play interactions, and examining the relationships between arousal, engagement, and play environment would be of value and could be used to inform development of engagement supports.

### Language across play settings

Although there was an interaction of play setting on our participants’ engagement, no such interaction was apparent with the number of spoken utterances children used. Our findings revealed there was no meaningful difference in how often children used spoken language across the symbolic and gross motor play settings. While no quantitative differences were observed, there may well have been qualitative differences across play settings that were not examined within our study. For example, the children might have used different communicative functions, different vocabulary, or syntactic constructions in the two settings and this should be explored in future work. Although there was little difference in the number of spoken utterances across the two different settings, this finding is noteworthy because this pattern differs from that observed in non-autistic children, who used far fewer spoken utterances when playing with gross motor toys, relative than when playing with symbolic toys (e.g., O'Brien & Bi, 1995). Therefore, our findings, although preliminary, should expand consideration of how play settings might be used in clinical settings when evaluating and working with young autistic children.

Future exploration of children’s utterances in relation to their engagement states merits exploration in order to provide a more complete characterization of language use during different play settings. Additionally, aligned with the transactional model of development, previous research has suggested that autistic children’s use of language is significantly associated with their caregivers’ communication (Fusaroli et al., 2019). We have yet to explore if there were differences in how the parents used language across the two play environments, but we acknowledge that this could have impacted our findings.

### Limitations

Although informative, this study is characterized by a number of limitations that should be considered. First, because we used previously collected videos, we were not able to alternate the order in which symbolic and gross motor toys were presented to the children. As such, our analysis is subject to bias in that the order of presentation could have impacted children’s overall stronger performance within the gross motor play setting. In future work, this could be addressed by randomizing order of the play settings.

It should also be noted that our data were extracted from 5-min samples for each play setting (10-min total). This duration is consistent with recommendations for engagement language samples (Miller, 1981). However, we do not know if this pattern would be sustained over a longer period of time (e.g. a 30-min therapy session). This should be taken into account when considering how to apply this information clinically. Future work could examine longer samples of play interactions to establish scalability.

Further, nonverbal communicative acts like gestures and non-word vocalizations were not coded and thus could not be analyzed. Given the age and language levels of our sample, examination of non-speaking communicative acts would have been beneficial and should be examined within future studies.

Additionally, although efforts were made to avoid bias in the sample selection when participants were recruited for the original study, self-selection bias was present. Parents who signed up for the original study from which the data was obtained had to make a considerable time commitment (17-h/week for 12months). They also reported higher than average education and income levels. Thus, participants might not be representative of the general population and thus limit generalizability of our findings.

Finally, and perhaps most importantly, this study did not explicitly consider the dyadic, bidirectional nature of social attention and communication, the impact that the play contexts may have had on caregiver language or interaction styles, and how these factors might interact with child outcomes. We know that children’s engagement and spoken language use is inextricably intertwined and dependent on their partner’s communication and actions. For example, caregiver quality of language and responsiveness have been shown to predict early language learning in neurotypical children (e.g., Hirsh-Pasek et al., 2015; Tamis–LeMonda et al., 2001) and autistic children (e.g., Haebig et al., 2013). Further, caregiver responsivity has been linked to the amount of time children jointly engage with their interaction partners (Patterson et al., 2014; Ruble et al., 2008). Although our engagement coding system (informed by Adamson et al., 2000) took into consideration the actions of both the caregiver and child, without systematic examination of caregivers’ contribution to this bidirectional interaction process, we only have a partial understanding of the impact of play contexts on autistic preschool children’s engagement and communication. Additionally, past research conducted with parents of non-autistic children have signaled that parents use different language patterns while playing with different types of toys (O’Brien & Nagle, 1987). Future work examining the impact of play settings on caregiver’s language and interaction styles and examination of how they mediate children’s engagement and communication is needed to gain a more complete picture.

### Clinical implications and significance

While our results warrant replication and expansion before concluding that one particular play setting is better than another for autistic preschoolers, our findings suggest there is value in clinicians differentiating play settings when assessing and supporting the social engagement capacities of young autistic children. It may be that specific elements within gross motor play settings provide some autistic children with important sensory-regulatory supports that positively impact their social engagement. Thus, if a child is having difficulty engaging with their play partners in a setting with symbolic toys, the clinician may want to explore where positive changes can be made in the child’s social engagement within a gross motor play setting. Furthermore, the recognition of a relationship between play setting and autistic children’s engagement states can help in the design of supports for autistic preschoolers. For example, when delivering a parent mediated social communication program, a clinician may recognize that a child naturally spends more time engaged with their caregiver when in a play setting with gross-motor toys. Therefore, the clinician designs a home practice plan that encourages the caregiver to use gross motor play settings to enhance the likelihood of success in socially engaging with their child, and the aim of promoting caregiver’s self-efficacy. Recognition of the relationship between gross motor play settings and autistic children’s social engagement may also encourage more interdisciplinary work between professionals who support social communication and sensory-motor domains (e.g., speech-language pathologists with occupational therapists, physical therapists, and recreation therapists). In future research, it will be important to explore why and for whom play settings are associated with engagement and language use is needed to be able to more accurately guide clinical practice.

## Conclusions

Findings from this sample of participants, using a broad coding scheme, illustrated a relationship between preschool-aged autistic children’s social engagement and their play settings (with gross motor toys vs. symbolic toys), and no relationship between the play settings and children’s number of spoken utterances. Our findings extend the existing literature by revealing *how* children engage differently across gross motor and symbolic play contexts – with the most significant findings being that children spent more time engaged with caregivers during gross motor based play contexts, and more time spent engaged with objects only when engaged in symbolic play contexts. Further research is needed to replicate findings, explore causal relations, and investigate factors that predict the impact of play setting on children’s engagement and communication, which could be used to inform clinical decision making and development of supports for autistic preschoolers. Moreover, the findings encourage us to continue to study how autistic children socially engage and use language when in play settings with exposure to different types of toys using a broader cross disciplinary lens, in hopes of better understanding how to support autistic children’s engagement and use of language.
